# A171 IL-23 RECEPTOR VARIANT PREDICTS ANTI-TNFα INDUCED PARADOXICAL PSORIASIS IN INFLAMMATORY BOWEL DISEASE

**DOI:** 10.1093/jcag/gwae059.171

**Published:** 2025-02-10

**Authors:** N Natt, M Gindi, A Wilson

**Affiliations:** Gastroenterology, Western University, London, ON, Canada; Gastroenterology, Western University, London, ON, Canada; Gastroenterology, Western University, London, ON, Canada

## Abstract

**Background:**

Anti-tumor necrosis factor (TNF)-α therapy is frequently used to treat inflammatory bowel disease (IBD). Paradoxical psoriasis (PP) is a known adverse effect of this drug class resulting in disfiguring skin lesions that impact quality of life and lead to treatment discontinuation. To date, there are no tools to identify individuals who are susceptible to developing PP. Variation in the IL-23 receptor (IL23R) gene has been linked to psoriasis onset and may be implicated in PP.

**Aims:**

To study the incidence and outcomes of PP in anti-TNFα treated IBD patients at an academic centre in London, Ontario and examine risk factors associated with PP including the IL23R variant (*IL23R1142G>A*).

**Methods:**

We performed a retrospective study of adult IBD patients treated with anti-TNFα therapies at London Health Sciences Centre between 2012 and 2024. Patient charts were reviewed for clinical variables and disease outcomes from the time of anti-TNFα exposure to treatment discontinuation or last follow-up. Patients’ serum was retrospectively genotyped for the *IL23R1142G>A* variant.

**Results:**

We identified 496 IBD patients with 570 unique anti-TNFα exposures between 2012 and 2024. 26 patients developed 29 cases of PP while the remaining 473 patients did not develop PP after exposure to 542 anti-TNFα therapies. There was an increased proportion of patients with PP who had a positive family and personal history of psoriasis, though the latter was not significant (Table 1). The IL23R variant was more common in patients who developed PP compared to those who did not (46.2% vs. 5.1%, OR 16.04, 95% CI 6.69-38.41, Figure 1). The average time to develop PP was 9.5 months, with 55% of patients receiving high-dose anti-TNFα drugs. Majority of patients had moderate-to-severe disease (72.4%) and required treatment cessation (69.0%). All participants had complete resolution of PP following interruption of anti-TNFα therapy.

**Conclusions:**

IL-23R mutations may identify those at risk for anti-TNFα induced PP. Personal and family history of psoriasis may also predict those at higher risk and should be elicited on assessment.

Table 1 - Baseline Characteristics

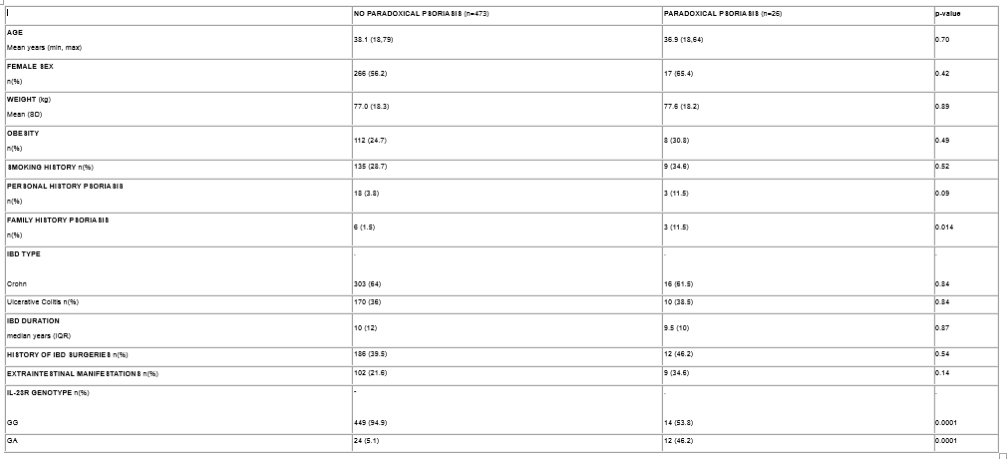

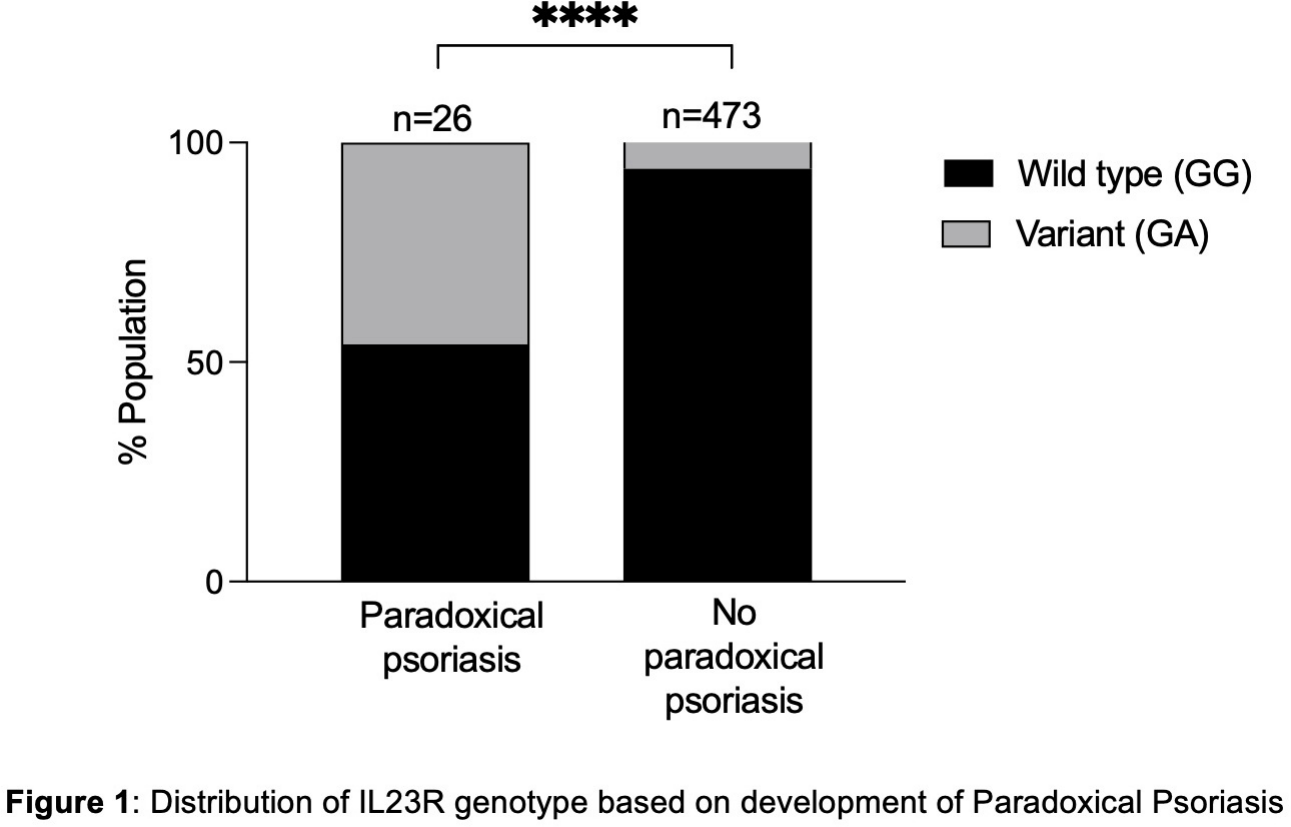

**Funding Agencies:**

None

